# CircVIS: a platform for circRNA visual presentation

**DOI:** 10.1186/s12864-022-08650-1

**Published:** 2022-06-09

**Authors:** Ya-Chi Lin, Yun-Chin Wang, Yueh-Chun Lee, Hui-Hsuan Lin, Kai-Li Chang, Yu-Chieh Tai, Kuei-Yang Hsiao

**Affiliations:** 1 Department of Plant Pathology, College of Agriculture and Natural Resources, National Chung Hsing University, Taichung, 40227 Taiwan; 2 Department of Medical Laboratory Science and Biotechnology, Asia University, Taichung, 41354 Taiwan; 3 Bachelor Program of Biotechnology, College of Agriculture and Natural Resources, National Chung Hsing University, Taichung, 40227 Taiwan; 4 Department of Radiation Oncology, Chung Shan Medical University Hospital, Taichung, 40201 Taiwan; 5 School of Medicine, Chung Shan Medical University, Taichung, 40201 Taiwan; 6 Program in Tissue Engineering and Regenerative Medicine, National Chung Hsing University, Taichung, 40227 Taiwan; 7 Department of Physiology, National Cheng Kung University, Tainan, 70101 Taiwan; 8 Department of Bioengineering, University of California, Berkeley, 94720 USA; 9 Institute of Biochemistry, College of Life Sciences, National Chung Hsing University, Taichung, 40227 Taiwan; 10 Program in Translational Medicine, College of Life Sciences, National Chung Hsing University, Taichung, 40227 Taiwan; 11 Rong Hsing Research Center for Translational Medicine, College of Life Sciences, National Chung Hsing University, Taichung, 40227 Taiwan

**Keywords:** Circular RNA, Subcellular localization, Reference transcript, Coding circRNA, Backsplice, Polysome, Polyribosome, Chromatin-associated

## Abstract

**Background:**

The collection of circRNAs mostly focused on their sequence composition such as protein/miRNA binding motif, and/or regulatory elements such as internal ribosome entry site. However, less attention was paid to subcellular localization. CircVIS aimed to provide a collection of circRNAs with information of subcellular compartments and also integrated the circRNA entries from previous circRNA databases.

**Results:**

A collection of circRNAs from public circRNA databases and de novo identification were annotated according to subcellular localizations including nucleoplasm, chromatin-associated parts, cytoplasm and polyribosome. All circRNAs were aligned to a selected major transcript, and if presence, the circRNA-derived open reading frame with annotation of functional domain were compared to its parental protein. The results showed that distinct circRNAs may exert their molecular and cellular functions in different subcellular compartments. The web service is made freely available at http://lab-x-omics.nchu.edu.tw/circVIS.

**Conclusions:**

CircVIS allows users to visualize the alignment between a given circRNA and its most relevant reference transcript along with information of subcellular localization.

## Background

Circular RNA (circRNA) is a novel class of single stranded regulatory RNA molecules with covalently enclosed ends by 3’, 5’-phosphodiester bond formed through backsplicing which takes place between a downstream splice donor and an upstream splice acceptor. Recent studies using next generation sequencing and computational analyses have revealed widespread existence of circRNAs in animals and many other organisms [1–3].

CircRNAs play various roles such as transcriptional activation, post-transcriptional modulation, translation and protein interaction in different subcellular compartments [4–8]. For those circRNAs regulating gene expression network through interaction with miRNAs [9–13], the majority of these circRNAs resides in cytoplasm to regulate the availability of miRNAs bound to mRNA molecules. For example, more than 75% of circular RNA originated from exon 8–10 of CCDC66, which interacted with miR-33b, 93 and 185, were found in the cytoplasm [11]. In contrast, it was reported that circRNAs modulating transcriptional activation associate with genomic DNA in nuclei. A few intron-retained circRNAs reside in the nuclei and associated with promoter region of target genes [5]. Nevertheless, an exonic circRNA from gene FLI1 modulating DNA methylation in promoter regions also localized in the nuclei [14]. These examples demonstrated that subcellular localizations of a given circRNA may provide clues to their molecular functions. Pioneer studies have made great contribution dissecting and archiving these relationships among miRNAs, circRNAs and associated pathological phenotypes [15–18]. However, the studies investigating the biological functions of circRNAs are largely limited to the function of miRNA sponge [19–21], and thus how to explore alternative molecular functions of circRNA become a critical task.

In this study, we analyzed and categorized circRNAs according to their subcellular localizations, aiming to provide more insight to interpret how circRNAs may exert their biological functions in distinct subcellular compartments. We also integrated potential coding region(s) along with functional domains of circRNA-derived open reading frames in a visual presentation platform.

### Implementation

#### Data retrieval and processing

**Fig. 1:**
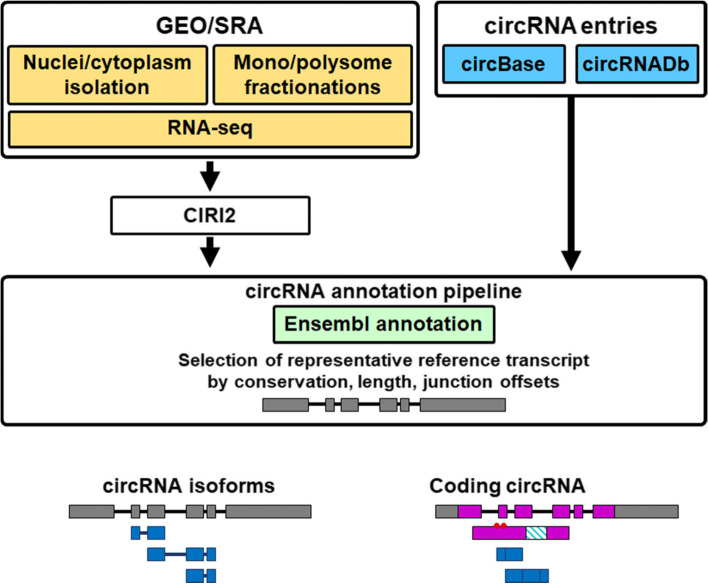
The schematic illustration of circRNA annotation tool. RNA sequencing data compatible with circRNA analysis from distinct subcellular compartments were retrieved from GEO/SRA databases (top-left corner). The coordinates of backsplicing junction were extracted by using CIRI2. The previously archived circRNA information were downloaded from circBase and circRNADb (top-right). Both sources of data were re-annotated by using gene information from Ensembl database. Each backsplicing junction was mapped to a selected representative reference transcripts according to conservation/number of exon/length of transcripts and alignment to exon junctions. CircRNAs will be displayed along with their reference transcript (bottom-left) and/or with the coding region from their parental genes (bottom-right)

The archived circular RNA coordinates were downloaded from circBase [22] and circRNADb [23] while raw data of RNA sequencing were directly downloaded from SRA and converted to fastq by using SRA tool kit (v 2.9.1). The dataset ‘SRP083953’ was used for ribosomal RNA-depleted cytoplasmic, nucleoplasmic and chromatin-associated RNA [24], while ‘SRP114807’ (all available fractions), ‘SRP139916’ (with cycloheximide treatment) and ‘SRP233220’ were used for polysome fractions [25, 26]. The read sequences were then aligned to reference genome (Homo sapiens GRCh38.92) using Burrows-Wheeler Aligner. For bisulfite treatment-derived samples (SRP233220), reads were aligned to the same reference genome with C converted to T or G converted to A. The resultant Sequence Alignment/Map files were then proceeded to CIRI2 (Fig. 1) [27].

#### Identification of circRNA representative transcript

The information of host genes and transcripts were extracted by comparison between circRNA coordinates and gene annotation (Homo sapiens GRCh38.92). Transcripts matched to circRNA coordinates were further ranked by the presence of Consensus Coding Sequence (CCDS), number of exons, offset to known exon junction and commonness among circRNAs.

#### Analysis of opening reading frame of circRNAs

**Fig. 2:**
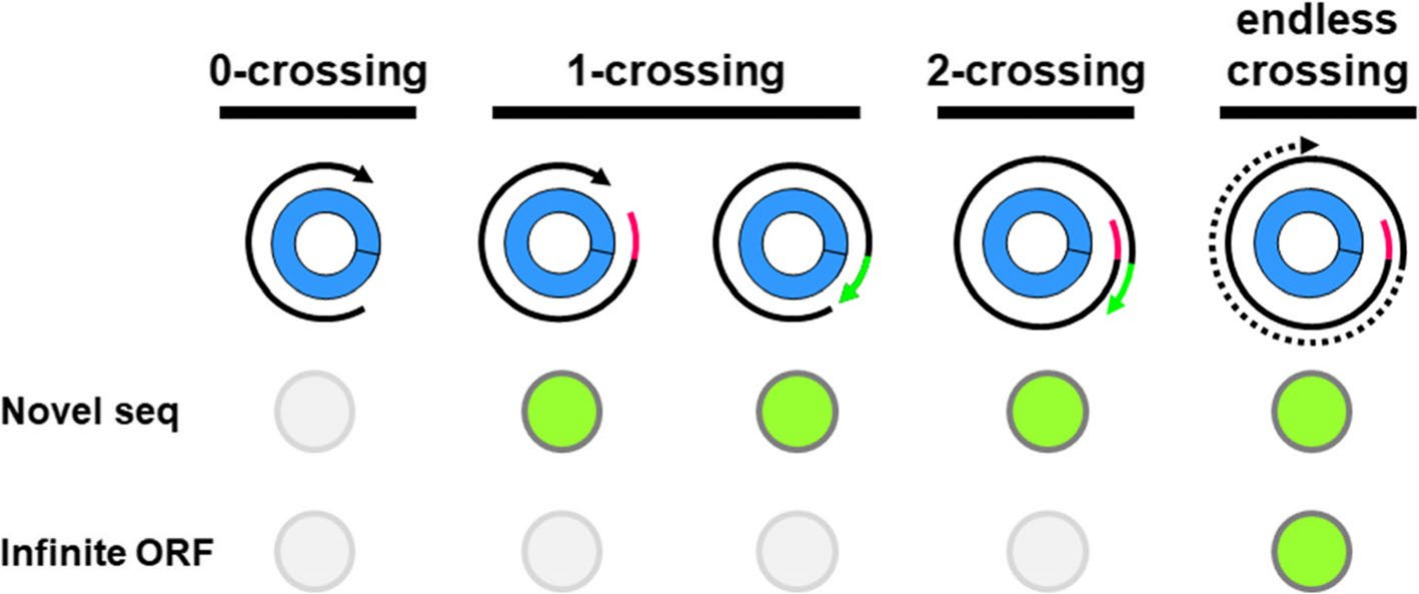
The analysis of ORFs in circular RNAs. The possible ORF organization were illustrated. The ORFs not crossing backsplicing junction were denoted ‘0−crossing’ (left). ORFs crossing backsplicing junctions (once) toward 5’ or 3’ will generate novel N terminal or C terminal sequences. 2C denoted the ORFs crossing backsplicing junction twice. In some cases, circRNA generates ORF crossing backsplicing junction infinite times. Grey circle denotes the absence of a feature (novel sequence or infinite ORF) while green circle denotes the presence of the feature

The potential ORFs of circRNA sequence were considered beyond its original length. According to the times of ORFs crossing backsplice junction, ORFs were classified to 0-crossing (0C), 1-crossing (toward 5’ or 3’: 1C5’, 1C3’), 2 crossings (2C) and endless crossing (edlsC) (Fig. 2). The zero-crossing ORFs are indistinguishable to ORFs in parental RNAs (Fig. 2, left), while 1C5’ or 1C3’ are ORFs with novel sequences in either N- or C-terminus. In a similar fashion, 2C ORFs would be expected to have novel sequences at both ends (Fig. 2, right). In some cases, endless crossing takes place, generating an ORF with infinite length.

#### Data visualization

The circRNA isoforms, corresponding host gene and information of the paired backsplicing exons were integrated and presented by using ‘ggplot2’ [28]. The circRNAs with open reading frame were extracted and aligned with the protein sequence of their parental gene. The functional annotation of the given protein was retrieved and presented by using functions adapted from ‘drawProteins’ with modifications [29].

## Results

### Genome-wide recollection of circular RNAs with distinct subcellular localizations

**Fig. 3:**
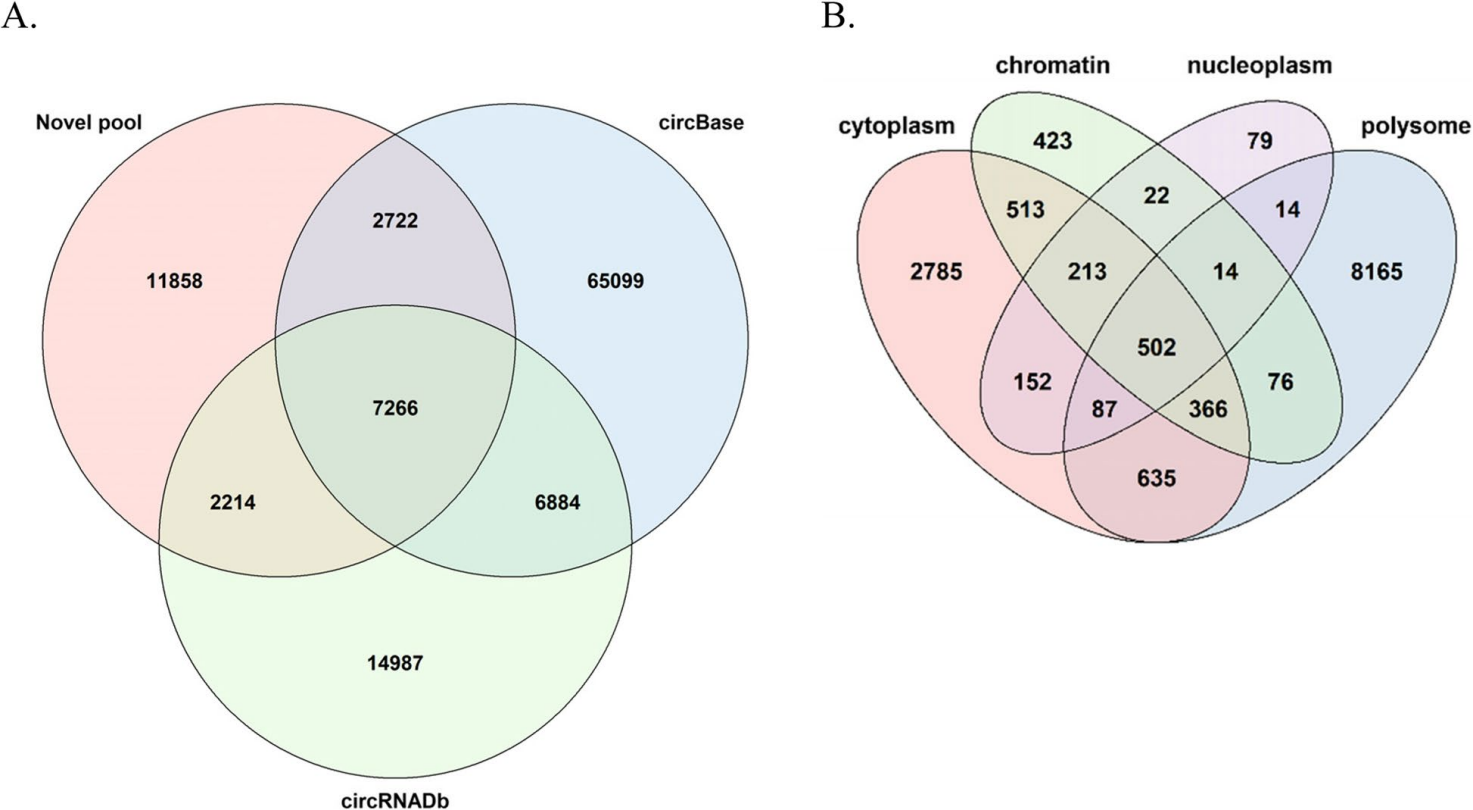
Genome-wide recollection of circRNAs. **A** A Venn diagram showed the overlapping unique entries among three datasets. The raw entries from circBase and circRNADb were preprocessed by selection of best representative transcript and paired exons for inter-database comparison. **B** The numbers of backsplicing events extracted from RNA-seq data using RNA collected from different cellular fractions were shown as a Venn diagram. In addition to the RNA isolated from the cytoplasmic and nuclear fractions, the chromatin fraction was defined as the insoluble parts of the nuclear lysate while the ‘polysome’ came from the heavy fractions of the sucrose gradient for polysome profiling

The comparison of the records between databases is important for biologists to design their experiments. Due to the incompatibility of accession ID, it is not intuitive to know whether a given circRNA in one database is present in the other. Our annotation pipeline assigned each circRNA a major reference transcript along with a pair of exons for backsplice, making comparison and communication easier. The results of comparison between circBase and circRNADb using our annotation pipeline demonstrated the feasibility of comparison. The pioneer databases of circRNA, circBase and circRNADb, shared 14 thousand circRNA entries, and there are 67 thousand circBase-specific and 17 thousand circRNADb-specific entries respectively. In comparison with circBase and circRNADb, the RNA-seq data we analyzed revealed additional unique 11,858 circRNAs which were not archived previously. Furthermore, we identified circRNAs residing in distinct subcellular localization. Our analyses revealed that the majority of circRNAs resides in cytoplasm based on HeLa and HCT116 cell-lines (Fig. 3B, cytoplasm and polysome, 11,585, 82.48%). Only limited number (524, 3.73%) of circRNAs locates in nuclei and/or associated with chromatin according to data from HeLa cells (Fig. 3B). Of special note, a previous study has found that depletion of particular proteins may impair the nuclear export of circRNAs in a size-dependent manner [30]. It will be worthwhile to further validate the correlation between the cellular distribution of circRNAs and these proteins.

### CircRNAs have great diversity of splicing pattern

**Fig. 4:**
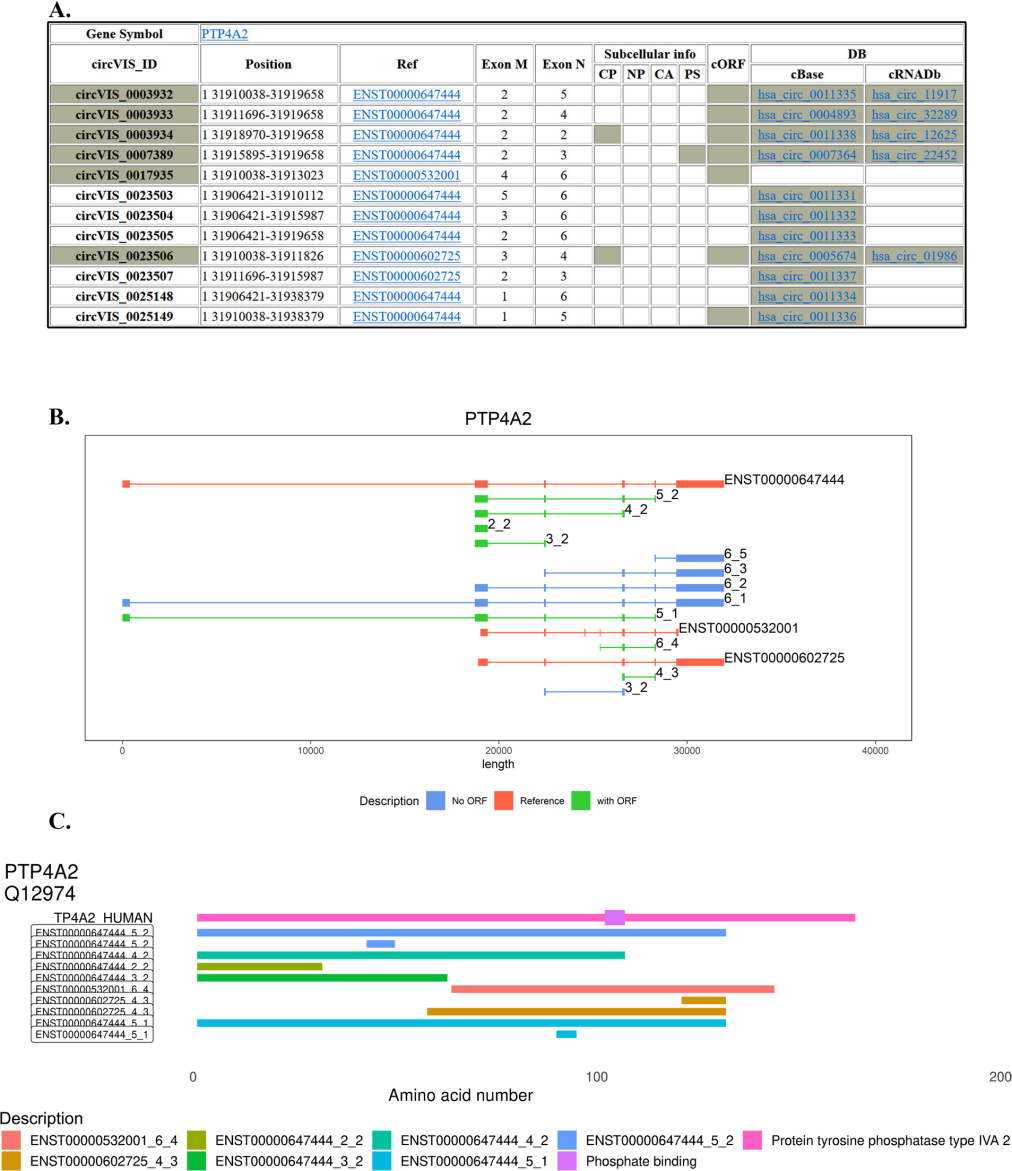
CircRNA isoform presentation. **A** The table shows details of the given circRNA including the origin of circRNA, Ref transcript, the pair of exon M/N for backsplice taking place from the representative transcript. The column of subcellular info shows ‘green’ if a given circRNA is identified in corresponding different subcellular compartments. Similarly, if ORF is predicted, the column of ‘cORF’ (circORF) shows green. The last two columns indicate in which database (DB) the given circRNA was archived (cBase: circBase; cRNADb: circRNADb). **B** Results of circRNA isoform presentation. The representative transcripts are showed in red tracks. A subset of circRNAs are aligned to the upper track (ENST00000647444) while others fit to ENST00000602725 or ENST00000532001. Green track: CircRNAs predicted to have ORF; Blue track: circRNA predicted not to have ORF. **C** CircRNAs with ORFs aligned to its protein with annotated domains. ORFs from different transcripts are color-coded. Paired exons of backsplicing exons are labeled at the end of gene symbol

To better compare all backsplice junctions from a single gene using minimal number of reference transcript, we integrated coordinates of backsplice junction, representative reference transcript, the pair of exons for backsplice, subcellular location and accession numbers of alternative circRNA databases (Fig. 4A). Furthermore to have better insight to this diversity, a proper visual aid is required to observe an overall exon usage for a given circRNA on a representative transcript. We integrated the annotations of Ensembl transcripts (red track) and circRNAs (blue or green track) in a single transcript plot (Fig. 4B). CircRNAs with predicted open reading frames can be easily identified. From the example of gene ‘PTP4A2’, the transcript ENST00000647444 had 9 pairs of backsplicing exons while ENST00000602725 and ENST00000532001 had 2 and 1 respectively. Obviously, one reference transcript can’t fit to all pairs of backsplicing exons. In addition, potential ORFs were aligned with parental proteins with functional features (Fig. 4C). While the functional domains were shown on the top of the reference protein, the circRNA-derived ORFs were aligned to the parental protein and shown at the bottom. This visual presentation will provide more information for biologists to evaluate what circRNAs may potentially modulate the functions of parental protein.

## Discussion

CircRNA is a class of regulatory molecules with diverse functions. Most of studies focused at its miRNA binding capacity in the cytoplasm. There are a few online resource designing for dissecting this function. For example, both starBase and circAtlas collected the interactions between circRNAs and miRNAs using either CLIP data or bioinformatic prediction while ACT classified the potential sponging activity through common target gene analysis [12, 17, 31]. In addition, predicted IRES and ORF information were archived in circRNADb and circAtlas [23, 31]. Nevertheless, CircInteractome archived the potential RNA-binding proteins associated with circRNA [32]. These annotations are extremely useful to promote functional studies for dissecting the particular downstream genes of circRNAs. However, the fact that overwhelming number of miRNA binding sites and ubiquitous presence of IRES/ORFs in databases of circRNA hinder the precise application of this information in biological researches. Thus, additional information has to be added to facilitate the analysis prior to experimental design.

The regulatory molecules exert their molecular functions in the corresponding subcellular compartments. For example, transcriptional factors such as ‘estrogen receptors’ (ERα and ERβ) or members of ‘signal transducer and activator of transcription’ (e.g. STAT3) have to translocate to nuclei to modulate the transcriptional activity of their target genes. In contrast, the majority of miRNAs and the associated AGO2 protein complexes resides in cytoplasm to target mRNA. Adherent to this concept, we hypothesized that the subcellular compartment where the circRNAs reside may provide extra information to predict or interpret their molecular functions. For example, circZNF609 [4], one of the best studied coding circRNAs was shown in polysome fraction in our analysis while circCCNB1 which modulates CDK1 activity [33] in nuclei was identified in nuclear fraction, suggesting that the information of subcellular localization indeed coincides with molecular functions in some cases. However, the limitation came from the variety of samples analyzed. First, there were limited number of available datasets compatible for circRNA analysis from multiple cell-lines. The majority of RNAseq data were from polyA-enriched samples, and/or oligo-dT-based library construction. Either one renders the circRNA analysis impossible. Second, there were limited number of RNA sequencing datasets available from subcellularly fractionated samples. Thus, the absence of circRNA in certain compartments will require further experimental evaluation.

## Conclusions

Our circRNA annotation platform not only provides a unique information about the subcellular location, but also a straightforward presentation and nomenclature. The integrative information is much improved compared to these pioneer databases (Table 1), and will serve as an alternative hub for circRNA studies.

**Table 1:** The comparison to other circRNA databases

	^**circVIS**^	^**circBase**^	^**circRNADb**^	^**circAtlas**^
^circRNA coordinates^	^hg38^	^hg19^	^hg19^	^hg19/hg38^
^Query using Gene symbol^	^●^	^●^	^●^	^Δ^
^Paired exon info^	^●^	^−^	^Δ^	^−^
^Representative transcript^	^●^	^●^	^●^	^−^
^To other Db^	^●^	^−^	^−^	^−^
^ORFs^	^●^	^−^	^Δ^	^●^
^Subcellular locations^	^●^	^−^	^−^	^−^
^Visual aid^	^●^	^Δ^	^−^	^●^
^Isoform presentation^	^●^	^●^	^−^	^−^

^**●**^^: available; −: not available; **Δ**^.^: incomplete^

## Availability and requirements

**Project name:** circVIS

**Project home page:** http://lab-x-omics.nchu.edu.tw/circVIS

**Operating system(s):** Platform independent (Web-based service)

**Programming language:** Perl 5 and R 3.5.0

**Other requirements:** Not applicable

**License:** GNU GPL; non-academic user: license needed

## Datasets used

circBase:http://www.circbase.org/

circRNADb:http://reprod.njmu.edu.cn/cgi-bin/circrnadb/index.php

SRA datasets:SRP083953, SRP114807, SRP139916, SRP233220

## Data Availability

Web service is made freely available at http://lab-x-omics.nchu.edu.tw/circVIS.

## Abbreviations

circVIS: CircRNA visual presentation
circRNA: Circular RNA
